# Analysis and Formation of Polycyclic Aromatic Hydrocarbons in Canned Minced Chicken and Pork during Processing

**DOI:** 10.3390/molecules29184372

**Published:** 2024-09-14

**Authors:** Baskaran Stephen Inbaraj, Yu-Wen Lai, Bing-Huei Chen

**Affiliations:** 1 Department of Food Science, Fu Jen Catholic University, New Taipei City 242062, Taiwan; sinbaraj@yahoo.com (B.S.I.); linda871123@gmail.com (Y.-W.L.); 2 Department of Nutrition, China Medical University, Taichung 404328, Taiwan

**Keywords:** PAHs, canned minced chicken, canned minced pork, QuEChERS, GC–MS/MS, PAH precursors

## Abstract

Polycyclic aromatic hydrocarbons (PAHs) represent important toxic compounds formed in meat products during processing. This study aims to analyze 22 PAHs by QuEChERS coupled with GC–MS/MS in canned minced chicken and pork during processing. After marinating raw minced chicken and pork separately with a standard flavoring formula used for canning meat in Taiwan, they were subjected to different processing conditions including stir-frying, degassing and sterilizing at 115 °C/60 min (low-temperature–long-time, LTLT) and 125 °C/25 min (high-temperature–short-time, HTST). The quantitation of PAHs in these meat products revealed the formation of only three PAHs including acenaphthylene (AcPy), acenaphthene (AcP) and pyrene (Pyr) in canned minced chicken and pork during processing with no significant difference in total PAHs between the meat types. Analysis of PAH precursors showed the presence of benzaldehyde at the highest level, followed by 2-cyclohexene-1-one and trans,trans-2,4-decadienal in canned minced chicken and pork, suggesting PAH formation through the reaction of benzaldehyde with linoleic acid degradation products and of 2-cyclohexene-1-one with C4 compounds through the Diels–Alder reaction, as well as the reaction of trans,trans-2,4-decadienal with 2-butene. Monounsaturated and polyunsaturated fatty acids were present in the largest proportion in LTLT-sterilized chicken/pork, followed by HTST-sterilized chicken/pork and raw chicken/pork, and their levels did not show a high impact on PAH formation, probably due to an insufficient heating temperature and length of time. A two-factorial analysis suggested that PAH formation was not significantly affected by the sterilization condition or meat type. Principal component analysis corroborated the observed results implying the formation of PAHs in canned minced chicken/pork under different processing conditions with an insignificant difference (*p* > 0.05) between them, with the individual PAH content following the order of Pyr > AcPy > AcP.

## 1. Introduction

Polycyclic aromatic hydrocarbons (PAHs) are composed of carbon, hydrogen and nitrogen atoms, with an amine group attached to the ring structure. They are often present in high-protein foods processed at high temperatures such as fried meat products. PAHs, a class of hydrophobic compounds with two or more aromatic rings, can be produced through the incomplete combustion and thermal degradation of organic matter, both of which are the main sources of PAHs found in the environment. Because of its stable chemical structure, PAHs do not easily decompose in nature and thus readily accumulate in the food chain [1,2]. At >200 °C, the combustion of carbon-containing organic compounds such as fat, protein, or carbohydrate may contribute to PAH formation, which is favorable at 500–900 °C. At a high temperature, both dehydrogenation and bond-breaking are the main reactions to proceed, while at a low temperature, the molecular polymerization reaction can proceed, leading to formation of PAH with a cyclic structure through the bending of carbon atoms [3,4]. The European Food Safety Authority has established four specific PAHs (PAH4) including BaA, CHR, BbF and BaP as indicators of carcinogenicPAHs in food [5], while the IARC [6] has classified BaP as being in Group 1 (carcinogenic to humans); CcdP, DBahA and DBalP are classified as Group 2A (probably carcinogenic to humans); Nap, BaA, CHR, MCH, BbF, BkFL, BjF, IP, DBaiP and DBahP are classified as Group 2B (possibly carcinogenic to humans); and Acp, FL, Phe, Ant, Flu, Pyr, BcF, DBaeP and BghiP are classified as Group 3 (not classified as human carcinogens), with the full names of all PAH compounds being shown in Table 1. Among the various PAHs, BaP (benzo [a]pyrene) is the most important one reported to be carcinogenic to humans [6]. It was shown that humans who are exposed to PAHs for a long period of time may suffer from lung cancer due to inhalation [7], gastric cancer caused by the ingestion of PAHs in food [8] and skin cancer because of skin contact [9]. In addition, pregnant women’s exposure to PAHs may result in a low intelligence quotient, as well as abnormal behavior and asthma in babies [10]. Thus, the reduction in exposure to PAHs is a vital issue in maintaining human health.

It has been well-documented that many factors including the food type, flavoring composition, oil type, cooking method and processing condition can affect the formation and reduction in PAHs in food/food products. In a review article by Singh et al. [11], different cooking methods and processing techniques such as heating, drying, baking, frying, roasting/toasting, grilling, barbecuing, smoking and ohmic-infrared cooking were shown to contribute to various PAHs being formed in processed food, with the level of PAHs depending on the distance from the heat source, fuel variety, degree of processing, cooking method, duration time and temperature. In meat/meat products, the formation of PAHs involves a complex mechanism influenced by many factors, with food components such as fat, protein and carbohydrates in meat products as well as processing conditions such as the processing method, temperature and time playing a pivotal role [12,13]. Although fat can enhance the unique flavor of meat products, its pyrolysis during thermal processing results in PAH formation. For instance, Kao et al. [14] showed that total PAHs were formed at higher levels in lamb stick (547.5 ng/g) and chicken thigh (118.3 ng/g) when compared to lean shrimp (42.6 ng/g) which could be associated with their fat contents at 11.96%, 9.06% and 0.2%, respectively. Likewise, a higher level of PAH formation was reported in air-fried chicken wings than in thigh and breast owing to their fat levels present at 14.9%, 2.8% and 1.2%, respectively [15]. Also, the oil type used for marinating and frying can significantly affect PAHs formation with the levels increasing following a rise in heating temperature and time length. Generally, the higher the degree of unsaturation of fatty acids, the greater the formation of PAHs, with several previous studies demonstrating this phenomenon in both model and food systems [16,17,18].

The PAH formation in meat products can also be affected by the content and type of proteins incorporated as additives, which can undergo pyrolysis and polymerization to produce PAHs. More specifically, free amino acids can react with reducing sugar via the Maillard reaction to generate key precursors for the formation of PAHs. In two different studies, cured beef satay and smoked dried fish containing 25.8% and 61.5–82.9% of amino acids were, respectively, shown to produce a 3-fold and 6-fold higher level of PAHs than beef satay and smoked fish with lower amino acid contents (10.0% and 47.1–79.2%) [19,20]. More recently, Lin et al. [18] demonstrated that aromatic amino acids can produce more PAHs than heterocyclic and aliphatic amino acids in a model system. In another study, the effect of 19 amino acids on PAH formation in beef patties was investigated and phenylalanine was shown to promote PAH formation, while lysine, aspartic acid, glutamic acid, valine and methionine could inhibit PAH formation [21]. Additionally, the pyrolysis of carbohydrates can lead to the deoxygenation of oxygenated aromatic compounds through the Diels–Alder reaction during high-temperature pyrolysis, resulting in the formation of PAHs in food. For instance, a study by Nie et al. [22] revealed that the level of BaP content in grilled sausages rose from 0.88 ng/g to 1.26 ng/g and 5.59 ng/g following the addition of D-fructose and D-glucose, respectively. Likewise, in a recent study Lin et al. [18] reported that the presence of water at pH 2–5 and heating at a temperature of 200 °C or 250 °C generated 2–6 ringed PAHs in a glucose model system, while the total PAH content increased following the elevation of the temperature to 300 °C as well as being accompanied by the generation of more carcinogenic PAHs. Thus, it is imperative to explore the role of food components and processing conditions on PAH formation in food/food products, especially in meat products.

Canning is a traditional method of food preservation through the complete destruction of microorganisms by heat, and thus most canned food products are commercially sterile and have a shelf life of two years or more. However, under high-pressure and high-temperature conditions, PAHs may be produced at a high content in meat products during canning. In a recent study, Tsao et al. [23] explored the effect of sterilization conditions on the formation of possible carcinogens, furan and its derivatives in canned foods including meat paste, tomato mackerel, chicken puree, tomato paste, pineapple slice, pineapple juice and carrot juice, and the contents of furan and its derivatives in these canned foods were shown to rise substantially. However, no information is available as to the formation of PAHs in canned meat products during processing. This study was thus undertaken to explore the formation of PAHs in canned minced pork and chicken, both of which are popular meat commodities on Taiwan’s market, during marinating, stir-frying, degassing and sterilization.

## 2. Materials and Methods

### 2.1. Materials

A total of 24 PAH standards including NaP, AcPy, AcP, Flu, Phe, Ant, FL, Pyr, BcF, Triphenylene (internal standard), BaA, CHR, MCH, BbF, BjF, CcdP, BaP, IP, DBahA, BghiP, DBalP, DBaeP, DBaiP and DBahP with purity at ≥98% were obtained from Sigma-Aldrich Co. (St Louis, MO, USA). The full names of the PAH standards are shown in Table 1. PAH precursor standards including 4,4-dimethyl-2-cyclohexene-1-one, 2-cyclohexene-1-one, cyclohexene, benzaldehyde and trans,trans-2,4-decadienal with purity ≥95% were also purchased from Sigma-Aldrich Co. A gas chromatograph (7890B)-mass spectrometer (5977A) equipped with an autosampler and headspace sampling unit as well as a DB-5 MS capillary column was obtained from Agilent Technologies (Palo Alto, CA, USA). The QuEChERS extraction and purification kits were from Yu-Ho Co (New Taipei City, Taiwan). The HPLC-grade solvents including methanol, acetonitrile, acetone and hexane were obtained from Merck (Darmstadt, Germany), while acetic acid was obtained from Sigma-Aldrich Co. Deionized water was produced using a Milli-Q water purification system from Millipore Co. (Bedford, MA, USA). Minced pork and lumpy chicken were purchased from a local supermarket located in New Taipei city, Taiwan. The lumpy chicken was further broken into pieces in a blender for subsequent experiments.

### 2.2. Processing of Canned Minced Pork and Chicken

**Figure 1:**
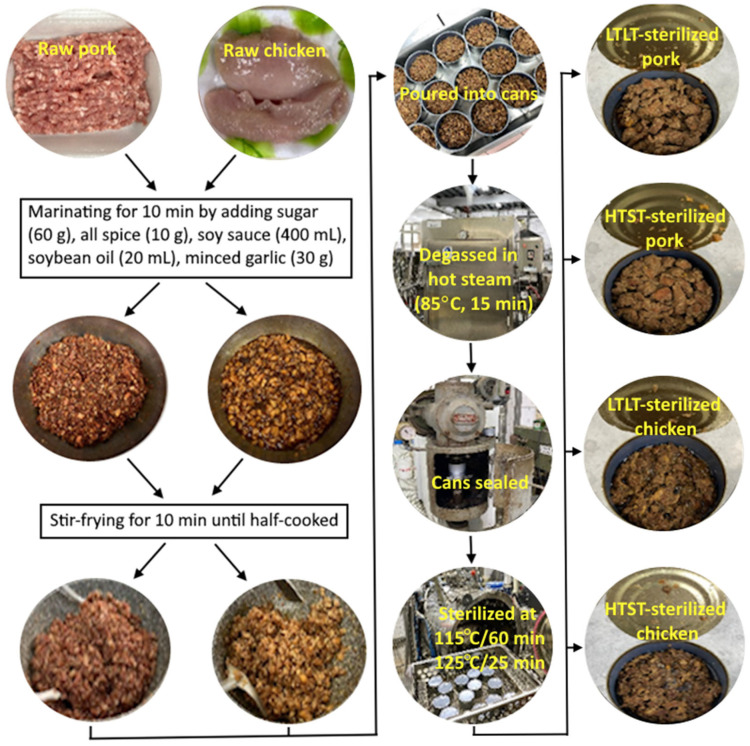
Processing steps for canned minced chicken and pork along with the four pictures in the last column showing the appearance of the products. A total of six samples obtained separately from raw, marinating, stir-fried, degassed, LTLT-sterilized and HTST-sterilized minced chicken/pork samples were analyzed in triplicate. LTLT-sterilized chicken/pork, low-temperature–long-time sterilized chicken/pork at 115 °C/60 min; HTST-sterilized chicken/pork, high-temperature–short-time sterilized chicken/pork at 125 °C/25 min.

The processing of canned minced pork and chicken was performed according to a method reported by Tsao et al. [23]. At first, 2 kg of minced pork or 2 kg of minced chicken was mixed separately with a flavoring formula consisting of sugar (60 g), allspice (10 g), soy sauce (400 mL), soybean oil (20 mL) and minced garlic (30 g). After stirring for 10 min to marinate, the mixture was poured into a pan for stir-frying (10 min). The above conditions were based on the standard pretreatment method used for preparing flavored minced pork and chicken for canning in Taiwan. Then, a total of 20 cans (307 × 201 mm No. 2 cans) were divided into 10 cans each for chicken and pork, followed by filling each can with a 150 g net weight of flavored minced meat, degassing with hot steam at 85 °C for 15 min and sealing. Of the 10 cans with flavored minced pork or chicken, 5 cans were sterilized in a retort at 115 °C/60 min (LTLT treatment), while the remaining 5 cans at 125 °C/25 min (HTST treatment). All 20 cans were then cooled to room temperature with cold water, followed by homogenizing, collecting a portion (2 g) of pork or chicken sample in duplicate and analyzing PAHs by QuEChERS coupled with GC–MS/MS. Figure 1 shows the processing steps and appearance of the as-processed products.

### 2.3. Basic Composition of Raw, Degassed and Canned Minced Chicken and Pork

According to the methods of the Chinese National Standards (CNS) of the Republic of China (Taipei, Taiwan), the basic composition of raw, degassed and canned minced chicken and pork including moisture, crude fat, crude protein and ash during processing was determined in duplicate [24,25].

### 2.4. Extraction and Purification of PAHs in Raw, Marinated, Stir-Fried, Degassed and Canned Minced Chicken/Pork

PAHs were extracted and purified in raw, marinated, stir-fried, degassed and canned minced chicken/pork based on the QuEChERS method described by Lai et al. [26]. Initially, 2 g of minced chicken/pork sample was mixed with deionized water (10 mL) in a centrifuge tube containing a ceramic homogenizer, followed by shaking (10 min) with 1% acetic acid in acetonitrile (10 mL), shaking (1 min) with 4 g magnesium sulfate and 1 g sodium acetate, and collecting the supernatant after centrifuging at 4 °C for 10 min (4000× *g*). For purification, the supernatant was mixed with PSA (300 mg), magnesium sulfate (900 mg) and C18 EC (300 mg), after which this mixture was shaken (1 min), centrifuged, the supernatant collected, dried under nitrogen and dissolved in hexane (0.2 mL) containing the internal standard Triphenylene (10 ng/mL) for PAH analysis by GC–MS/MS.

### 2.5. Analysis of PAHs in Raw, Marinated, Stir-Fried, Degassed and Canned Minced Chicken/Pork by GC–MS/MS

A total of 23 PAHs standards and the internal standard Triphenylene as well as the PAHs in raw, marinated, stir-fried, degassed and canned minced chicken/pork were separated within 77 min using a DB-5MS capillary column (30 m × 0.25 mm ID, film thickness 0.25 μm) in splitless mode with He as a carrier gas, flow rate at 1.25 mL/min, injector temperature at 320 °C and MS interface temperature at 280 °C with the following temperature programming condition: 80 °C in the beginning, maintained for 1 min; raised to 200 °C at 5 °C/min, maintained for 10 min; raised to 220 °C at 5 °C/min, maintained for 5 min; raised to 230 °C at 1 °C/min, maintained for 10 min; and raised to 320 °C at 10 °C/min and maintained for 10 min [26]. The retention time and SRM detection parameters are shown in Table 1.

### 2.6. Method Validation of PAHs in Raw Chicken/Pork

Based on a study reported by Lai et al. [26], the method validation of PAHs was performed by taking freeze-dried pork as the representative sample. Briefly, a total of 10 concentrations (0.03, 0.05, 0.1, 0.3, 0.5, 0.7, 0.9, 1, 3 and 5 ng/mL) of each PAH standard were prepared in hexane and added to a blank meat sample matrix for injection into the GC–MS/MS. However, the LOQ of pyrene was determined by preparing the same concentrations in hexane and injecting into the GC–MS/MS, as a meat sample matrix without pyrene could not be found. Then, both the limit of detection (LOD) and limit of quantitation (LOQ) were determined based on a signal/noise ratio (S/N) ≥ 3 and S/N ≥ 10, respectively. The recovery was determined by adding 10 ng/g and 50 ng/g of each PAH standard to freeze-dried pork separately in a 50 mL centrifuge tube, followed by extraction, purification and injection into the GC–MS/MS. The recovery of individual PAHs was then obtained based on the ratio of the detected amount of the standard relative to the amount of standard added. For the determination of precision, the intra-day variability analysis was performed by adding various PAH standards with a concentration of 10 ng/g each to freeze-dried pork for extraction, purification and injection into the GC–MS/MS separately for a total of 9 analyses on the same day with 3 replicates in the morning, afternoon and evening, while the inter-day variability analysis was carried out by following the same procedure with a total of 9 analyses on 3 consecutive days with 3 replicates each day [26].

PAH quantitation was conducted by subjecting freeze-dried pork samples to extraction and purification by QuEChERS initially and, after evaporating 1 mL of supernatant to dryness, 5 concentrations (10, 30, 50, 70 and 100 ng/mL) of various PAH standards were added to dried meat sample extract and injected into the GC–MS/MS for obtaining matrix-matched calibration curves and linear regression equations with R^2^. However, as pyrene was found to be present in freeze-dried pork, pyrene quantitation was conducted by preparing a total of 6 concentrations (0.1, 0.3, 0.5, 10, 30 and 50 ng/mL) of pyrene in hexane and injecting them into the GC–MS/MS to obtain the standard calibration curve [26].

### 2.7. Determination of PAH Precursors in Raw, Marinated, Stir-Fried, Degassed and Canned Minced Chicken/Pork by GC–MS

Initially, a 0.5 g sample of chicken/pork was poured into a 20 mL headspace vial containing water (2.5 mL), followed by heating (65 °C for 10 min), inserting fiber head (65 μm PDMS/DVB, Supelco, PA, USA) into sample vial, heating again (65 °C for 20 min) and inserting into the Agilent GC–MS inlet for desorption into the injection port (260 °C for 1 min) with a splitless mode. The separation of PAH precursors was conducted in an Agilent HP-5MS column (30 m × 0.25 mm × 0.25 um film thickness) with a helium carrier gas flow rate at 1 mL/min and the temperature programming was 40 °C initially and maintained for 4 min; raised to 50 °C at 5 °C/min and held for 2 min; increased to 120 °C at 5 °C/min and held for 3 min; and finally increased to 260 °C at 30 °C/min and maintained for 5 min. The mass spectrometer was operated in the electron-ionization (EI) mode at an ionization voltage of 70 eV with SIM mode being used for their identification and quantification at *m*/*z* 82, 68, 67, 105 and 81, respectively, for 4,4-dimethyl-2-cyclohexene-1-one, 2-cyclohexene-1-one, cyclohexene, benzaldehyde and trans,trans-2,4-decadienal. Then, stock solutions of these standards (1000 µg/mL each) were prepared in methanol, followed by preparing calibration curves in the concentrations ranging from 0.1 to 20 ng/mL for 4,4-dimethyl-2-cyclohexene-1-one and 2-cyclohexene-1-one each as well as 0.6 to 20 ng/mL for cyclohexene, 0.1 to 800 ng/mL for benzaldehyde and 4 to 100 ng/mL for trans,trans-2,4-decadienal. Finally, the quantitation of each precursor in raw, marinated, stir-fried, degassed and canned minced chicken/pork was performed based on the linear regression equation obtained from the respective calibration curve [26].

### 2.8. Fatty Acid Composition Analysis in Raw and Canned Minced Chicken/Pork by GC

Initially, fat was extracted from raw chicken/pork and canned minced chicken/pork using the Soxhlet extraction method [27] for subsequent analysis of the fatty acids in raw and canned minced chicken/pork by GC based on a report by Chou et al. [28]. Then, a 0.5 g oil sample was collected and mixed with 10 mL of potassium hydroxide solution in methanol (0.5 N), after which this mixture was heated in a water bath (90 °C) for 10 min for saponification. Next, 14% boron trifluoride in methanol (8 mL) was added after cooling, followed by heating in a water bath (90 °C) for 5 min, adding hexane (8 mL), heating at 90 °C for 3 min, cooling, adding sodium chloride solution (60 mL) and separating into two layers. The upper layer was collected and added with anhydrous sodium sulfate (0.2 g), after which hexane was removed under vacuum, diluted with hexane (10 mL) and 1 μL injected into the GC with the injector temperature at 250 °C, column temperature at 220 °C, detector temperature at 280 °C (flame ionization detector) and flow rate at 4 mL/min with He as the carrier gas and the split ratio at 1:40. An Agilent DB-WAX high-polar polyethylene glycol capillary column (60 m × 0.53 mm ID, 1 μm film thickness) with an Agilent 6890 GC instrument (Palo Alto, CA, USA) was used. The various fatty acids in oil samples were identified by comparing the retention times of the standards with peaks on the chromatogram and the peak area of each fatty acid calculated as a percentage for quantitation based on total percentage of fatty acids.

### 2.9. Statistical Analysis

A total of six samples obtained separately from raw, marinated, stir-fried, degassed, LTLT-sterilized and HTST-sterilized minced chicken/pork samples were analyzed in triplicate. Then, all the data were subjected to statistical analysis for an analysis of variance (ANOVA) and Duncan’s multiple range test for comparison of the statistical significance of the mean values at *p* < 0.05 by the statistical analysis software system (SAS) (version 6, SAS Institute Inc, Gary, NC, USA) [29], while principal component analysis (PCA) was performed by Origin^®^ 2019b version 9.65 (OriginLab Corporation, Northampton, MA, USA).

#### 2.9.1. Factorial Analysis

To determine the independent contributions of meat type and sterilizations (115 °C/60 min and 125 °C/25 min) and their interactions (meat type × sterilization conditions), a two-way ANOVA-based factorial analysis was performed by elucidating if the mean data of independent groups and their interactions were statistically significant or not.

#### 2.9.2. PCA

The mean PAH content data obtained for raw, marinated, stir-fried, degassed, LTLT-sterilized and HTST-sterilized minced chicken and pork samples were used to run PCA by grouping the PAH contents formed under various treatments and transferring a set of correlated variables into a fresh set of linearly uncorrelated variables based on an eigen value > 1. To learn the differences and similarities between different processing conditions involved in the canning of minced chicken and minced pork, PCA was performed with a Kaiser–Meyer–Olkin value of 0.80 and *p* < 0.05. The various treatment data used for PCA include the PAHs formed in unprocessed raw meat, marinated minced meat, stir-fried minced meat at 95 °C for 10 min, degassed minced meat at 85 °C for 15 min, LTLT-sterilized meat at 115 °C for 60 min and HTST-sterilized meat at 125° for 25 min.

## 3. Results and Discussion

### 3.1. Basic Composition of Raw, Degassed and Canned Minced Chicken and Pork

The basic composition including the moisture, ash, crude fat and crude protein contents of raw minced chicken and pork as well as their degassed, LTLT-sterilized and HTST-sterilized samples during the canning process varied from 70.88 ± 0.91% to 74.04 ± 0.13%, 1.15 ± 0.08% to 3.34 ± 0.01%, 2.17 ± 0.00 to 2.27 ± 0.02% and 22.56 ± 0.08 to 23.51 ± 0.06% in minced chicken, respectively, while in minced pork, it ranged from 68.12 ± 0.35% to 71.37 ± 0.24%, 0.98 ± 0.003 to 2.94 ± 0.003%, 12.05 ± 0.01% to 12.45 ± 0.18% and 15.53 ± 0.17% to 16.80 ± 0.12%. Comparatively, the moisture, ash and protein contents were higher in raw, degassed and canned minced chicken, while the crude fat contents were higher in raw, degassed and canned minced pork.

### 3.2. Analysis of PAHs by GC–MS/MS in Raw, Marinated, Stir-Fried, Degassed and Canned Minced Chicken/Pork

**Figure 2:**
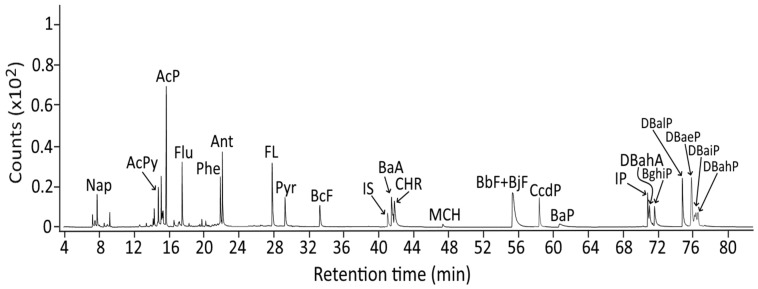
Chromatograms of 24 PAH standards in SRM mode detected by GC–MS/MS. PAH, polycyclic aromatic hydrocarbons; GC–MS/MS, gas chromatography–tandem mass spectrometry; SRM, selected reaction monitoring; IS, internal standard (Triphenylene).

Following the extraction and purification condition described in the Methods section, PAHs were extracted by QuEChERS from raw, marinated, stir-fried, degassed and canned minced chicken/pork for subsequent analysis by GC–MS/MS. A total of 24 PAH standards including the internal standard Triphenylene were separated within 77 min with retention times ranging from NaP (7.90 min) to DBahP (76.8 min) (Figure 2, Table 1). The precursor ions (*m*/*z* 128–276) and product ions for the confirmation (*m*/*z* 78–274) as well as the precursor ions (*m*/*z* 128-302) and product ions (*m*/*z* 102-300) for quantitation of 22 PAHs are also shown in Table 1. However, we have to point out that method validation was not carried out as it was performed with PAH standards and freeze-dried raw pork in a previous study by Lai et al. [26]. In brief, among the 23 PAHs, the LOD and LOQ ranged from 0.03 ng/g (DBalP) to 0.5 ng/g (BaP) for the former and 0.1 ng/g (DBalP) to 1.5 ng/g (BaP) for the latter, with the same LOD of 0.1 ng/g and LOQ of 0.3 ng/g for all three of the PAHs (AcPy, ACP and Pyr) found in raw, marinated, stir-fried, degassed and sterilized chicken/pork samples in this study [26]. Moreover, the coefficient of variation (CV) for the intra-day variability ranged from 5.03 to 10.57% and 6.74 to 15.6% in standards and freeze-dried raw pork for 22 PAHs, respectively, as well as from 9.12 to 17.25% and 11.91 to 20.61% for the inter-day variability [26]. These data meet the acceptable CV limits set by the TFDA for intra-day variability at <30% and inter-day variability at <32% for analyte concentrations ranging from 0.001 to 0.01 ppm [30]. Similarly, the recovery of 22 PAHs ranged from 84.6 to 107.6% in the standards and 80.1 to 101.1% in freeze-dried raw pork [26], which conforms to the acceptable recovery range (60–125% for 10 ppb and 70–120% for 50 ppb) recommended by the TFDA [30].

### 3.3. PAH Content Changes in Canned Minced Chicken and Pork during Processing

**Figure 3:**
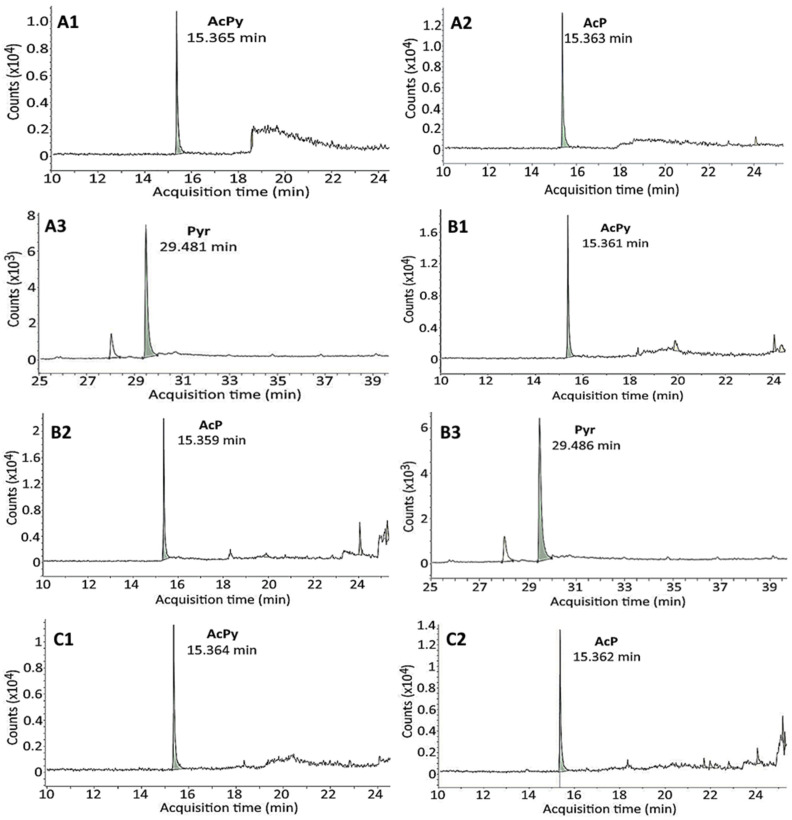

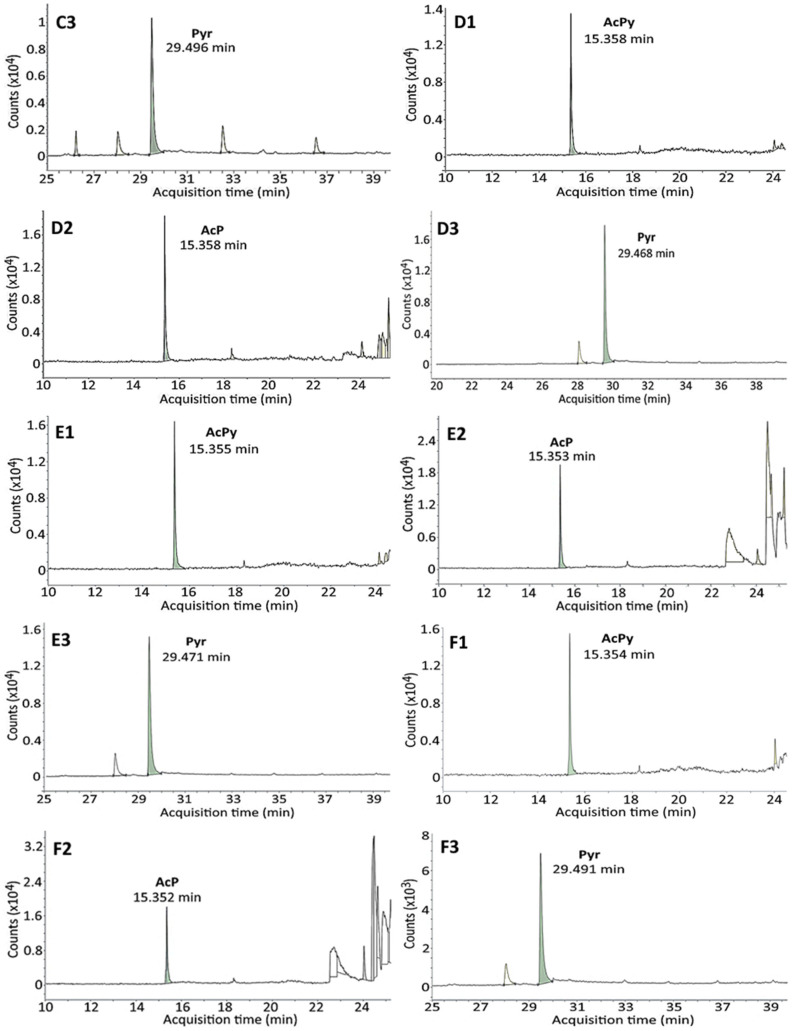

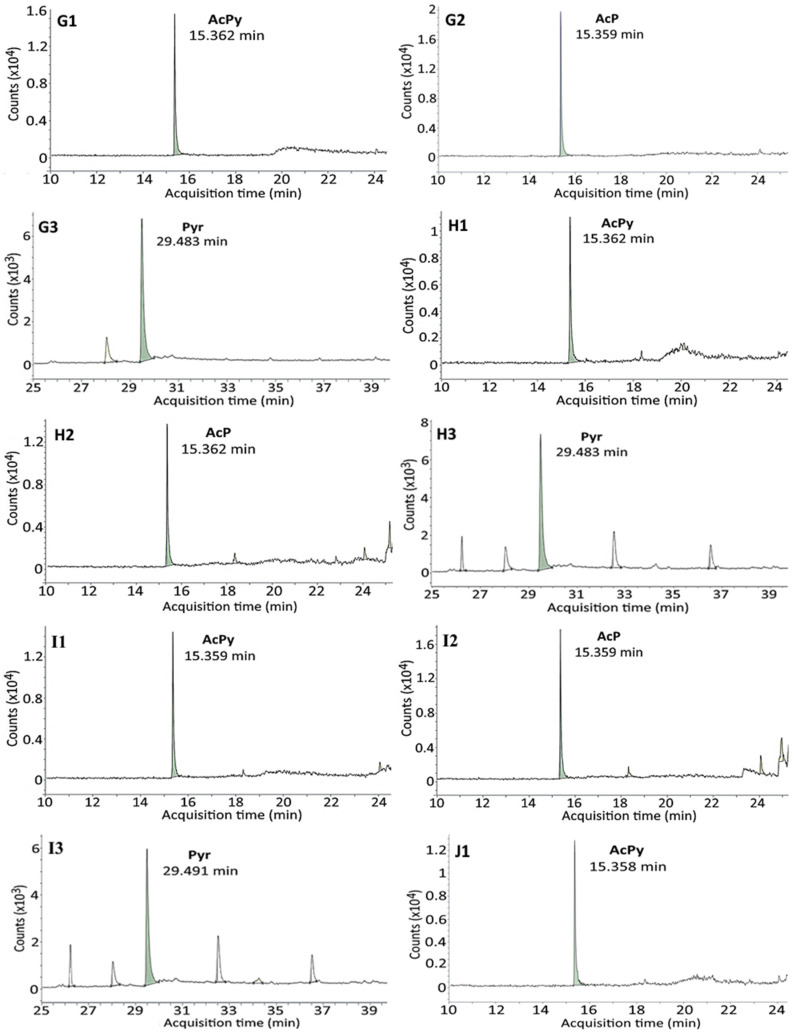

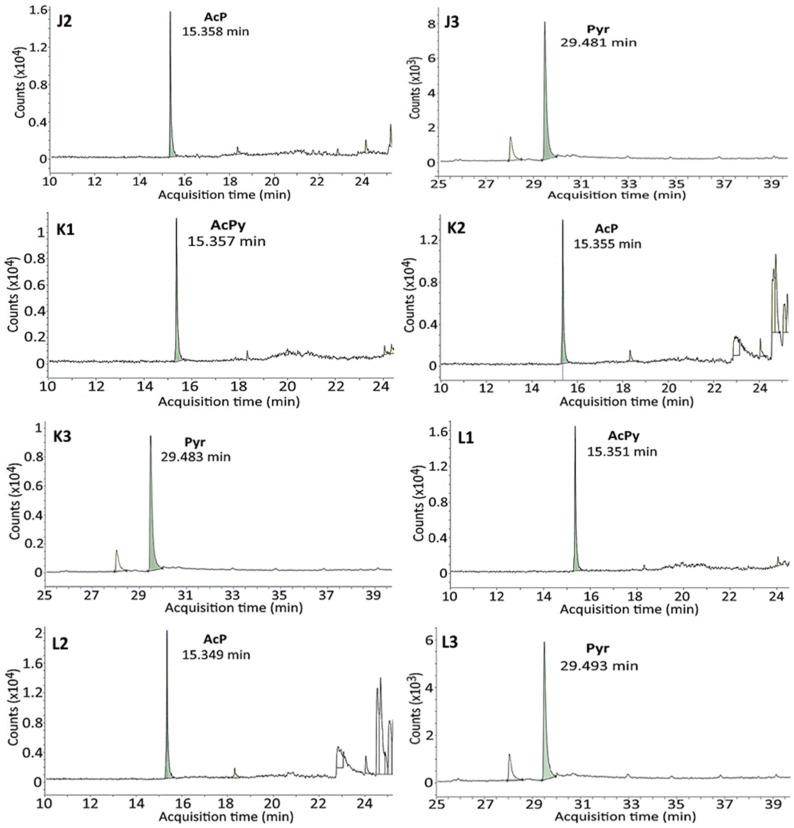
GC–MS/MS (SRM mode) chromatograms of PAHs in canned minced chicken (**A**–**F**) and minced pork (**G**–**L**) as affected by different processing conditions including raw chicken (**A1**–**A3**) and pork (**G1**–**G3**), marinated minced chicken (**B1**–**B3**) and pork (**H1**–**H3**), stir-fried minced chicken (**C1**–**C3**) and pork (**I1**–**I3**) at 95 °C for 10 min, degassed minced chicken (**D1**–**D3**) and pork (**J1**–**J3**) at 85 °C for 15 min, low-temperature–long-time (LTLT) sterilized canned minced chicken (**E1**–**E3**) and pork (**K1**–**K3**) at 115 °C for 60 min, high-temperature–short-time (HTST) sterilized canned minced chicken (**F1**–**F3**) and pork (**L1**–**L3**) at 125° for 25 min. GC–MS/MS, gas chromatography–tandem mass spectrometry; SRM, selected reaction monitoring.

Figure 3 shows the GC–MS/MS chromatograms for the PAHs formed in raw, marinated, stir-fried, degassed and canned minced chicken and pork during processing, and the contents are presented in Table 2. Only three PAHs including AcPy (2.12 and 2.50 ng/g), AcP (1.54 and 1.70 ng/g) and Pyr (14.27 and 14.18 ng/g) were detected in raw chicken and pork, which may be associated with environmental factors such as water, air and soil. As reported by the WHO [31], PAHs are ubiquitous in the environment and their accumulation, migration and transformation can lead to entrance into the agriculture and food chain. Following marinating, only a minor change in the contents of AcPy, AcP and Pyr was shown in both chicken and pork. A similar outcome was observed in canned minced chicken after stir-frying, degassing, LTLT-sterilization and HTST-sterilization treatments. This outcome revealed that the heating temperature and time length may be inadequate to cause chemical changes in AcPy, AcP and Pyr, all of which possess a high stability during heating. Nevertheless, AcPy was produced at a higher level than AcP for all of the treatments, which should be due to the oxidation of AcP to AcPy in the presence of soy sauce [32]. Moreover, the total PAHs in stir-fried, degassed, LTLT-sterilized and HTST-sterilized chicken were, respectively, 19.17, 19.45, 20.68 and 20.85 ng/g, as well as 19.45, 19.96, 21.00 and 21.04 ng/g in pork with the same treatments. Apparently, there was no significant difference (*p* > 0.05) in the total PAHs between chicken and pork for stir-frying, degassing, LTLT-sterilization and HTST-sterilization treatments. However, a slight increase in the level of individual PAHs, especially AcPy and AcP, during the degassing and sterilization processing steps was shown which may be caused by prolonged heating (15–60 min). 

Currently, there is a lack of data regarding the formation of PAHs in canned minced chicken and pork during processing. Most studies focus on the determination of PAHs in canned fish and shellfish. For instance, Drabova et al. [33] analyzed the PAH contents in 54 samples of canned smoked and non-smoked fish and seafood products purchased from the Czech market and reported that PAHs were detected in all samples from 1.4 to 116 ng/g, with the highest level found in canned smoked sprats. In another study, El Morsy et al. [34] measured the PAH contents in canned tuna and sardines from different origins randomly sampled from supermarkets, and the total PAH contents were from 0.01 to 9.78 ng/g and from 1.56 to 2.69 ng/g, respectively. Additionally, the PAH contents were shown to be from 7.17 to 13.20 ng/g in imported frozen fish including *Megalaspis cordyla*, *Ctenopharyngodon idella*, *Labeo rohita* and *Acanthopagrus latus*, and from 12.25 to 28.61 ng/g in imported canned fish including sardines, mackerel and tuna based on a report by Al-Abdul-Nebi et al. [35]. Interestingly, the levels of five-ring PAHs and six-ring PAHs detected in canned mussels in a kimchi marinade containing vegetable oil, vinegar, paprika, salt and spices were lower than those in a natural pickling sauce containing water, salt and spices, probably caused by PAH migration from the mussels into the kimchi sauce [36]. It is also possible that PAHs can be absorbed by aquatic organisms present in the environment, leading to bioaccumulation in the marinade. As there is no significant change (*p* > 0.05) in PAH contents in canned minced chicken and pork during processing in our study, the presence of PAHs in these products should mainly be from the marinade ingredients.

### 3.4. PAH Precursor Content Changes in Canned Minced Chicken and Pork during Processing

The PAH precursor content changes in canned minced chicken and pork are also shown in Table 3. A total of three PAH precursors were identified, including 2-cyclohexene-1-one, benzaldehyde and trans,trans-2,4-decadienal, in all the heated chicken and pork samples, with benzaldehyde present at the highest level, followed by 2-cyclohexene-1-one and trans,trans-2,4-decadienal. It may be postulated that 2-cyclohexene-1-one can be oxidized to benzene for subsequent reaction with C4 compound to generate naphthalene through the Diels–Alder reaction. Also, benzaldehyde may react with linoleic acid degradation products containing conjugated diene such as 1,3-butadiene from edible oil to produce PAHs with polycyclic ring during heating. For trans,trans-2,4-decadienal, it may react with 2-butene to form 4-pentyl-2,3-dimethyl benzoic acid for subsequent reaction with 2-butene, leading to naphthalene generation.

Comparatively, in chicken and pork samples, the HTST sterilization contributed most to formation of these PAH precursors, followed by the LTLT sterilization, degassing, stir-frying and marinating. Compared to the LTLT-sterilization treatment, the HTST-sterilization treatment was shown to be more susceptible to PAH precursor formation. Nevertheless, a large increase in benzaldehyde content during sterilization did not result in a pronounced rise in the total PAH content as shown in Table 2, which may be due to the low sterilization temperature. Also, in addition to lipid oxidation, some other PAH formation mechanisms may be involved, as it was reported that a temperature > 200 °C is required to promote PAH formation through dehydrogenation and acetylene addition reaction [37].

### 3.5. Composition of Fatty Acid in Raw Chicken/Pork and Canned Minced Chicken/Pork

The composition of fatty acids in raw chicken/pork and canned minced chicken/pork are shown in Table 4. A much higher content of fat was shown in raw and sterilized pork than in raw and sterilized chicken. In raw, LTLT-sterilized and HTST-sterilized chicken, polyunsaturated fatty acids (PUFAs) accounted for 0.30%, 0.66% and 0.59%, respectively, as well as 0.48%, 0.58% and 0.49% for monounsaturated fatty acids (MUFAs), and 0.35%, 0.48% and 0.37% for saturated fatty acids, respectively. A similar trend was shown in raw, LTLT-sterilized and HTST-sterilized pork. By comparison, both MUFAs and PUFAs constituted the largest portion in LTLT-sterilized chicken/pork, followed by HTST-sterilized chicken/pork and raw chicken/pork. Under the same sterilization condition, a higher content of PAH precursors such as benzaldehyde and trans,trans-2,4-decadienal was generated in pork with a higher level of PUFAs when compared with chicken. However, the PAH formation remained slightly affected possibly due to a different mechanism involved as mentioned above. Thus, all three of the PAHs detected in the raw and canned chicken and pork samples belonged to the IARC Group 3 category representing ‘not carcinogenic to humans’ [6]. For PAH4 (BaA, CHR, BbF and BaP), in which BaP belongs to IARC Group 1 (carcinogenic to humans), while BaA, CHR and BbF belong to Group 2B (possibly carcinogenic to humans), with maximum permissible levels at 5.0 ng/g for BaP and 30.0 ng/g for PAH4 in heat-treated meat products [6,38]. As PAH4 remained undetected in canned minced chicken and pork in our study, the consumption of both products should be quite safe.

### 3.6. Two-Way ANOVA Factorial Analysis 

A two-factorial analysis on PAH formation as affected by sterilization treatment and meat type was performed using the two-way ANOVA method, and the results are shown in Table 5. The interaction between sterilization treatment and meat type showed an insignificant impact on PAH formation in canned minced chicken and pork (*p*-value, 0.89), as evident by a *p*-value of 0.82 for sterilization treatment and 0.60 for meat type. This outcome is in agreement with the above discussion of the results shown in Table 2, suggesting that the PAH formation was not significantly affected by the sterilization condition or meat type.

### 3.7. Principal Components Analysis (PCA)

**Figure 4:**
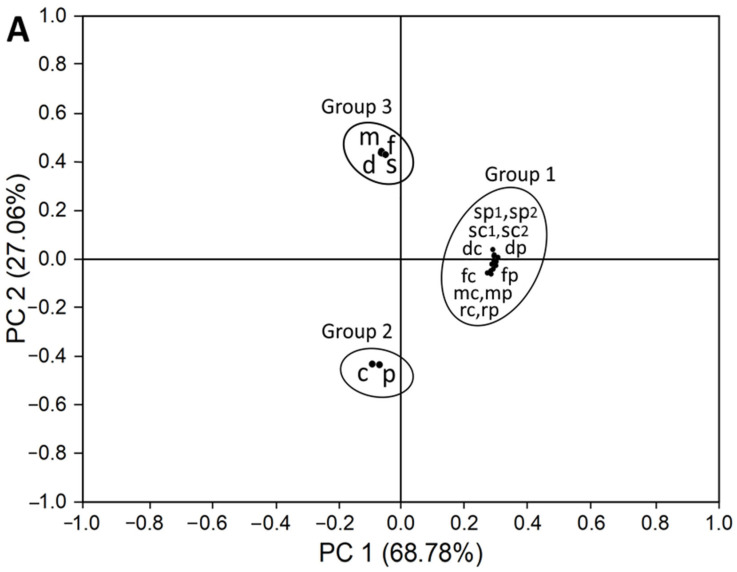

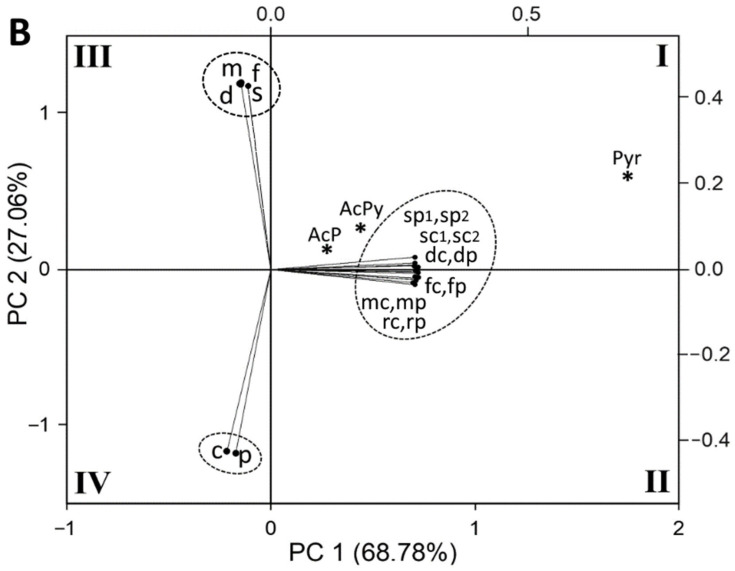
Principal component analysis for PAHs formation in canned minced chicken and minced pork as affected by different processing conditions with the plots showing the score plot (**A**) and the biplot consisting of a loading plot and score plot (**B**). PAHs, polycyclic aromatic hydrocarbons; rc and rp, the amount of PAHs formed in unprocessed raw chicken and raw pork; mc and mp, the amount of PAHs formed in marinated chicken and pork; fc and fp, the amount of PAHs formed in stir-fried chicken and pork; dc and dp, the amount of PAHs formed in degassed chicken and pork; sc1 and sc2, the amount of PAHs formed in sterilized chicken at 115 °C/60 min and 125 °C/25 min; sp1 and sp2, the amount of PAHs formed in sterilized pork at 115 °C/60 min and 125 °C/25 min; c and *p*, the amount of PAHs formed in chicken and pork regardless of processing condition; m, f, d and s, the amount of PAHs formed, respectively, in marinated meat, stir-fried meat, degassed meat and sterilized meat at 115 °C/60 min plus 125 °C/25 min regardless of meat type. The dark dot (●) symbol denotes principal component data for the formation of PAHs in chicken or pork under the above processing conditions. The asterisk (*) symbol represents the principal component data of the individual PAHs formed under the above processing conditions.

A principal component analysis for the formation of PAHs in canned minced chicken and pork as affected by different processing treatments is shown in Figure 4. Based on an eigenvalue of the correlation matrix > 1 at 12.38 and 4.87, two principal components including PC 1 and PC 2 with 68.78% and 27.06% were obtained, respectively, contributing to the maximum total variation of 95.84% for the studied conditions. Figure 4A shows the score plot illustrating that the principal component data obtained using the experimental mean contents of PAHs can be clustered into three groups depending on the degree of separation of data points in four quadrants. Group 1 represents various PAHs formed at 12 different treatments including unprocessed raw chicken (rc) or raw pork (rp), marinated chicken (mc) or pork (mp), stir-fried chicken (fc) or pork (fp), degassed chicken (dc) or pork (dp), LTLT-sterilized chicken (sc1) or pork (sp1) at 115 °C/60 min and HTST-sterilized chicken (sc2) or pork (sp2) at 125 °C/25 min. The closeness of the data points in Group 1 implied a slight variation in PAH formation under the studied processing conditions, which was well corroborated with the total PAH contents ranging from 17.93 to 20.85 ng/g and 18.38 to 21.04 ng/g, respectively, in chicken and pork (Table 2). Regardless of the processing condition, the total amount of PAHs formed in chicken and pork was clustered into Group 2, with the proximity of two data points revealing a slight variation in PAH formation between chicken and pork. Finally, the PCA data points representing the total amount of PAHs formed at different processing conditions including marinating (m, 18.76–19.13 ng/g), stir-frying (f, 19.17–19.45 ng/g), degassing (d, 19.45–19.96 ng/g) and LTLT/HTST sterilization conditions (s, 20.68–21.04 ng/g) regardless of meat type were closely located in Group 3 suggesting an insignificant difference in PAH formation under these processing conditions (Table 2).

Figure 4B shows the biplot containing both loading and score plots with a larger degree of angle between the loading plots representing a higher variation or smaller correlation in the PAHs formed under a specific processing condition. The smaller degree of angle between the loading plots corresponding to the mean PAH content data for raw chicken and pork as well as that for the canned minced chicken and pork shown in Group 1 implied a less formation of PAHs during the processing of canned minced chicken and pork with a slight difference in levels between them. Also, a smaller degree of angle for the loading plots in Group 2 confirmed the minor difference in total PAH formation between canned minced chicken and pork regardless of the processing conditions. Similarly, the loading plots for the total PAHs formed for ‘m’, ‘f’, ‘d’ and ‘s’ in Group 3 overlapped with a negligible or zero-degree angle of separation, suggesting again an insignificant difference in PAH formation between the marinating, stir-frying, degassing and sterilization conditions.

Furthermore, the score plots of individual PAH formation in raw chicken and pork as well as in canned minced chicken and pork under different processing conditions are also shown in Figure 4B with three asterisk symbols in quadrants I and II denoting that the formation of Pyr, AcPy and AcP highly impacted PC 1 and the position of the asterisk approaching the vertical zero line at the center suggesting their level of formation in the following order: Pyr > AcPy > AcP. This trend is in agreement with the row-wise total content of each PAH compound regardless of the meat type and processing condition as shown in Table 2. Consequently, the biplot in Figure 4B provides an overall grouping and correlation for PAH formation in chicken and pork as affected by different processing conditions. Overall, the PCA confirmed the formation of PAHs in canned minced chicken and pork under different processing conditions with an insignificant difference between them.

## 4. Conclusions

The formation of PAHs in canned minced chicken and pork was successfully determined by QuEChERS coupled with GC–MS/MS as affected by different processing conditions including marinating, stir-frying, degassing, LTLT sterilization (115 °C/60 min) and HTST sterilization (125 °C/25 min). Three PAHs (AcPy, AcP and Pyr) were generated in canned minced chicken/pork during processing and an insignificant change in total PAHs was shown between chicken and pork, with a concomitant rise in the level of PAH precursors including benzaldehyde, 2-cyclohexene-1-one and trans,trans-2,4-decadienal, implying the formation of PAHs by a different mechanism. A slight increase in the level of individual PAHs, especially AcPy and AcP, during the degassing and sterilization processing steps was shown which may be caused by prolonged heating. PCA also confirmed the formation of PAHs in canned minced chicken/pork under different processing conditions with an insignificant difference between them and the individual PAH content followed the order of Pyr > AcPy > AcP. Thus, for PAH4 (BaA, CHR, BbF and BaP), in which BaP belongs to IARC Group 1 (carcinogenic to humans) while BaA, CHR and BbF belong to Group 2B (possibly carcinogenic to humans), all remained undetected in the canned minced chicken and pork in our study, implying that the consumption of these products should be safe.

## Figures and Tables

**Table 1:** Retention time and SRM detection parameters of 23 PAHs and an internal standard (Triphenylene) by GC–MS/MS.

PAH Compound	Retention Time (min) ^a^	Quantitative Ion Pair		Qualitative Ion Pair	
		Precursor Ion > Product Ion	Collision Energy	Precursor Ion > Product Ion	Collision Energy
		(*m*/*z*)	(eV)	(*m*/*z*)	(eV)
Naphthalene (NaP)	7.90	128 > 102	20	128 > 78	25
Acenaphthylene (AcPy)	14.5	152 > 151	20	152 > 150	35
Acenaphthene (AcP)	15.6	154 > 153	20	153 > 152	20
Fluorene (Flu)	17.6	166 > 165	20	165 > 164	25
Phenanthrene (Phe)	21.8	178 > 176	35	178 > 152	25
Anthracene (Ant)	22.1	178 > 176	35	178 > 152	25
Fluoranthene (FL)	27.9	202 > 200	40	202 > 201	25
Pyrene (Pyr)	29.5	202 > 200	40	202 > 201	25
Benzo [c]fluorene (BcF)	33.6	216 > 215	20	215 > 213	30
Triphenylene (IS)	41.1	228 > 226	30	113 > 112	10
Benzo [a]anthracene (BaA)	42.0	228 > 226	35	113 > 112	15
Chrysene (CHR)	41.6	228 > 226	35	228 > 227	20
5-methylchrysene (MCH)	47.5	242 > 241	40	242 > 239	15
Benzo [b]fluoranthene (BbF)	55.8	252 > 250	40	125 > 124	15
Benzo [j]fluoranthene (BjF)	55.8	252 > 250	40	125 > 124	15
Cyclopenta [c,d]pyrene (CcdP)	58.3	226 > 224	45	113 > 112	15
Benzo [a]pyrene (BaP)	61.2	252 > 250	20	125 > 124	40
Indeno [1,2,3-cd]pyrene (IP)	70.8	276 > 274	45	137 > 136	15
Dibenzo [a,h]anthracene (DBahA)	71.0	278 > 276	40	276 > 274	45
Benzo [ghi]perylene (BghiP)	71.6	276 > 274	45	138 > 137	15
Dibenzo [a,l]pyrene (DBalP)	74.9	302 > 300	40	150 > 149	20
Dibenzo [a,e]pyrene (DBaeP)	75.9	302 > 300	40	150 > 149	20
Dibenzo [a,i]pyrene (DBaiP)	76.5	302 > 300	40	150 > 149	20
Dibenzo [a,h]pyrene (DBahP)	76.8	302 > 300	40	150 > 149	20

^a^ Retention time is based on GC–MS conditions described in Section 2.5. PAHs, polycyclic aromatic hydrocarbons; GC–MS/MS, gas chromatography tandem mass spectrometry; SRM, selected reaction monitoring; *m*/*z*, mass-to-charge ratio; IS, internal standard.

**Table 2:** Contents (ng/g) of PAHs in canned minced chicken and pork during processing ^1^.

	Raw	Marinated	Stir-Fried (95 °C/10 min)	Degassed (85 °C/15 min)	Sterilized (115 °C/60 min)	Sterilized (125 °C/25 min)
Chicken						
AcPy	2.12 ± 0.23 ^g^	2.66 ± 0.24 ^defg^	2.37 ± 0.08 ^fg^	3.12 ± 0.57 ^bcde^	3.72 ± 0.44 ^ab^	3.27 ± 0.46 ^bcd^
AcP	1.54 ± 0.06 ^e^	1.77 ± 0.17 ^cde^	1.74 ± 0.06 ^de^	2.04 ± 0.29 ^bcd^	2.34 ± 0.34 ^ab^	2.25 ± 0.13 ^abc^
Pyr	14.27 ± 0.05 ^c^	14.32 ± 0.03 ^c^	15.06 ± 1.07 ^ab^	14.28 ± 0.06 ^c^	14.61 ± 0.50 ^bc^	15.33 ± 0.67 ^a^
Total	17.93 ± 0.24 ^e^	18.76 ± 0.42 ^cde^	19.17 ± 1.08 ^cde^	19.45 ± 0.92 ^bcd^	20.68 ± 1.16 ^ab^	20.85 ± 0.16 ^a^
Pork						
AcPy	2.50 ± 0.49 ^efg^	2.56 ± 0.44 ^defg^	2.96 ± 0.24 ^cdef^	3.51 ± 0.37 ^abc^	3.75 ± 0.27 ^ab^	4.10 ± 0.42 ^a^
AcP	1.70 ± 0.33 ^de^	1.81 ± 0.41 ^cde^	2.07 ± 0.19 ^bcd^	2.32 ± 0.25 ^ab^	2.60 ± 0.23 ^a^	2.73 ± 0.39 ^a^
Pyr	14.18 ± 0.07 ^c^	14.75 ± 0.45 ^abc^	14.41 ± 0.18 ^bc^	14.14 ± 0.06 ^c^	14.64 ± 0.13 ^abc^	14.22 ± 0.09 ^c^
Total	18.38 ± 0.90 ^de^	19.13 ± 1.30 ^cde^	19.45 ± 0.56 ^bcd^	19.96 ± 0.66 ^abc^	21.00 ± 0.61 ^a^	21.04 ± 0.88 ^a^

^1^ Data are presented as the mean ± standard deviation of triplicate determinations, and data with different small letters (a–g) in the same row are significantly different (*p* < 0.05). PAH, polycyclic aromatic hydrocarbon; AcPy, acenaphthylene; AcP, acenaphthene; Pyr, pyrene.

**Table 3:** Contents (ng/g) of PAH precursors in canned minced chicken and pork during processing ^1^.

	Raw	Marinated	Stir-Fried (95 °C/10 min)	Degassed (85 °C/15 min)	Sterilized (115 °C/60 min)	Sterilized (125 °C/25 min)
Chicken						
4DCH	nd	nd	nd	nd	nd	nd
2CH	nd	12.60 ± 4.89 ^d^	25.92 ± 1.08 ^c^	39.55 ± 1.90 ^b^	38.21 ± 5.94 ^b^	49.09 ± 11.80 ^a^
CH	nd	nd	nd	nd	nd	nd
BAL	nd	17.72 ± 1.91 ^h^	82.67 ± 5.46 ^ef^	73.04 ± 6.20 ^f^	175.80 ± 10.39 ^c^	205.79 ± 17.24 ^b^
TTD	nd	nd	3.44 ± 0.15 ^de^	3.71 ± 0.10 ^cd^	3.92 ± 0.15 ^c^	3.95 ± 0.11 ^c^
Total	nd	30.32 ± 6.53 ^f^	112.03 ± 6.25 ^d^	116.30 ± 4.24 ^cd^	217.94 ± 4.61 ^b^	258.83 ± 14.88 ^a^
Pork						
4DCH	nd	nd	nd	nd	nd	nd
2CH	nd	7.54 ± 1.00 ^de^	25.22 ± 4.12 ^c^	24.47 ± 1.54 ^c^	24.69 ± 1.67 ^c^	27.24 ± 0.50 ^c^
CH	nd	nd	nd	nd	nd	nd
BAL	7.14 ± 1.06 ^hi^	39.11 ± 4.03 ^g^	95.56 ± 11.56 ^de^	103.30 ± 12.36 ^d^	223.80 ± 8.06 ^a^	234.37 ± 8.88 ^a^
TTD	3.68 ± 0.29 ^cd^	3.06 ± 0.05 ^e^	3.25 ± 0.08 ^e^	3.80 ± 0.08 ^cd^	8.43 ± 0.58 ^a^	7.85 ± 0.31 ^b^
Total	10.82 ± 1.34 ^g^	49.70 ± 4.93 ^e^	124.03 ± 15.37 ^cd^	131.57 ± 11.99 ^c^	256.92 ± 9.24 ^a^	269.46 ± 8.40 ^a^

^1^ Data are presented as the mean ± standard deviation of triplicate determinations and data with different small letters (a–i) in the same row for each precursor in both chicken and pork are significantly different (*p* < 0.05). 4DCH, 4,4-dimethyl-2-cyclohexene-1-one; 2CH, 2-cyclohexene-1-one; CH, cyclohexene; BAL, benzaldehyde; TTD, trans,trans-2,4-decadienol; nd, not detected.

**Table 4:** Composition and percentage of fatty acids in raw chicken/pork and canned minced chicken/pork.

Fatty Acids	Chicken			Pork		
	Raw	Sterilization		Raw	Sterilization	
		105 °C/60 min	125 °C/25 min		105 °C/60 min	125 °C/25 min
Saturated fatty acid						
14:0	nd ^c^	nd	nd	0.12	0.15	0.13
16:0	0.27	0.34	0.27	2.44	2.97	2.56
18:0	0.08	0.14	0.1	1.28	1.52	1.34
Total	0.35	0.48	0.37	3.84	4.64	4.03
trans fatty acid	nd	nd	nd	nd	nd	nd
cis-MUFA ^a^						
9c-16:1	nd	nd	nd	0.2	0.22	0.21
9c-18:1	0.48	0.58	0.49	4.2	4.92	4.25
11c-18:1	nd	nd	nd	0.25	0.29	0.25
11c-20:1	nd	nd	nd	0.08	0.1	0.08
Total	0.48	0.58	0.49	4.73	5.53	4.79
cis-PUFA ^b^						
18:3 ω3 (ALA)	nd	0.06	0.05	0.08	0.14	0.11
18:2 ω6	0.3	0.6	0.54	1.51	2.25	1.87
20:2 ω6	nd	nd	nd	0.06	0.07	0.06
20:4 ω6	nd	nd	nd	0.06	0.06	0.05
Total	0.3	0.66	0.59	1.71	2.52	2.09
Total fatty acid	1.13	1.72	1.45	10.28	12.69	10.91

^a^ MUFA, monounsaturated fatty acid; ^b^ PUFA, polyunsaturated fatty acid; ^c^ nd, not detected.

**Table 5:** A two-factorial analysis of PAH formation in canned minced chicken and pork as affected by meat type and the sterilization condition.

Factor	DF	SS	MS	F-Value	*p*-Value
Sterilization condition	1	0.035	0.035	0.05	0.82
Meat type	1	0.189	0.189	0.30	0.60
Sterilization condition × Meat type	1	0.012	0.012	0.02	0.89

DF, degree of freedom; SS, sum of squares; MS, mean squares.

## Data Availability

The data presented in this study are available in this article.
